# Lysine Methylation Modulates the Interaction of Archaeal Chromatin Protein Cren7 With DNA

**DOI:** 10.3389/fmicb.2022.837737

**Published:** 2022-03-03

**Authors:** Niannian Ding, Yuanyuan Chen, Yindi Chu, Cheng Zhong, Li Huang, Zhenfeng Zhang

**Affiliations:** ^1^State Key Laboratory of Microbial Resources, Institute of Microbiology, Chinese Academy of Sciences (CAS), Beijing, China; ^2^College of Life Sciences, University of Chinese Academy of Sciences, Beijing, China; ^3^The Research Platform for Protein Sciences, Institute of Biophysics, Chinese Academy of Sciences (CAS), Beijing, China; ^4^State Key Laboratory of Food Nutrition and Safety, Tianjin University of Science & Technology, Tianjin, China

**Keywords:** hyperthermophilic archaea, post-translational modification, lysine methylation, chromatin protein, Cren7, epigenetic regulation

## Abstract

Cren7 and Sis7d, two chromatin proteins from *Sulfolobus islandicus*, undergo extensive methylations at multiple lysine residues to various extents. Whether this highly conserved protein serves an epigenetic role in the regulation of the structure and function of the chromosome remains unclear. In the present study, we show that methylation significantly affects Cren7, but not Sis7d, in the ability to bind DNA and to constrain negative DNA supercoils. Strikingly, methylated Cren7 was significantly less efficient in forming oligomers or mediating intermolecular DNA bridging. Single-site substitution mutation with glutamine reveals that methylation of the four lysine residues (K24, K31, K42, and K48) of Cren7 at the protein-DNA interface, which are variably conserved among Cren7 homologues from different branches of the Crenarchaeota, influenced Cren7-DNA interactions in different manners. We suggest that dynamic methylation of Cren7 may represent a potential epigenetic mechanism involved in the chromosomal regulation in crenarchaea.

## Introduction

In all cellular organisms, the genomic DNA is packaged into a highly compressed and ordered structure found in nucleus of Eukaryotes or nucleoid of Bacteria and Archaea by a set of chromatin proteins (Dame et al., 2020; Chen et al., 2021; Laursen et al., 2021). A large number of studies have firmly established the importance of post-translational modifications (PTMs) of chromatin proteins to chromosome remodeling and epigenetic regulation. It is well known that PTMs, including acetylation, methylation, phosphorylation, ubiquitination, and biotinylation, on the N-terminal tail of histones from eukaryotes are involved in a number of cellular processes such as gene expression regulation and DNA damage response (Kouzarides, 2007; Zhao and Garcia, 2015). Among the PTMs of histones, dynamic methylation is considered to serve a key role in epigenetic regulation as it has been reported not only in the regulation of gene transcription (Shilatifard, 2008) but also in the formation of heterochromatin (Martin and Zhang, 2005).

As in Eukarya, PTMs of proteins are widely found in Archaea (Soppa, 2010; Gong et al., 2020). For example, lysine methylation has been reported for proteins involved in DNA transcription, DNA repair, cell division, cell cycle regulation and signal transduction, suggesting that this type of PTM may be of physiological significance to the growth and survival of Archaea (Vorontsov et al., 2016). However, to the best of our knowledge, no PTMs have been reported for archaeal histones, which generally lack the eukaryotic-like N-terminal tails. This is consistent with the wide absence of the homologs of eukaryotic protein methyltransferases and demethylases in Archaea (Henneman et al., 2018). Interestingly, two *Sulfolobus* chromatin proteins, Cren7 and Sul7d, have been shown to undergo dynamic methylation at multiple lysine residues to various extents (Baumann et al., 1994; Guo et al., 2008; Cao et al., 2018). The modification is catalyzed by aKMT, a non-SET domain protein lysine methyltransferase conserved in the Crenarchaeota (Chu et al., 2012). aKMT is a major protein methyltransferase responsible for the lysine methylation of the majority of proteins in *Sulfolobus islandicus* (Chu et al., 2016).

Cren7 is highly conserved in crenarchaea (Guo et al., 2008). It binds double-stranded DNA in the minor groove, inducing a severe DNA bending toward the major groove (Zhang et al., 2010), and thus folds DNA into an S-shape chromatin fiber (Zhang et al., 2019). The protein protects DNA duplex from thermal denaturation, constrains negative DNA supercoils and compacts DNA efficiently *via* bending and bridging *in vitro* (Guo et al., 2008; Zhang et al., 2020). Of the 12 lysines of Cren7 from *S. islandicus*, 9 were found to be methylated, among which K16 underwent both mono- and dimethylation (Cao et al., 2018). All of the modified residues (K5, K6, K9, K11, K16, K24, K31, K42, and K48) are solvent-exposed on the surface of Cren7. K24 and K31 have direct contacts with DNA, and K42 and K48 extend the side chains toward DNA from the edge of the DNA interface (Zhang et al., 2010, 2015). Sul7d, the most studied archaeal chromatin protein found only in *Sulfolobus* species, resembles Cren7 in both biochemical properties and 3-D structure (Robinson et al., 1998; Napoli et al., 2002; Zhang et al., 2020). Several lysines are dynamically methylated, and the extent of methylation depends on growth temperature (Baumann et al., 1994). Sis7d, a Sul7d family member from *S. islandicus* contains 14 lysine residues. Six of them (K5, K7, K9, K40, K53, and K61) were found to be methylated (Cao et al., 2018). Although these modified lysines are not directly involved in DNA interactions (Agback et al., 1998; Robinson et al., 1998), methylation of the protein was found to be affected by bound DNA in an *in vitro* assay (Niu et al., 2013).

Recently, Johnson et al. (2019) obtained three *S. solfataricus* strains with a heritable trait of super acid resistance, called SARC, through adaptive laboratory evolution (Payne et al., 2018). These strains acquired heritable conserved transcriptomes, leading to changes in both the phenotype and expression of the acid resistance genes, and one of them contained no mutations. Interestingly, a heritable alteration in the PTM pattern of Cren7 and Sso7d, a protein of the Sul7d family from *S. solfataricus*, was observed in all SARC lines. Both of the chromatin proteins were consistently undermethylated, whereas other chromatin proteins were unaltered (Johnson et al., 2019). The bulk of Sso7d was undermethylated at lysine residues (K5, K7, and K9) in the N-terminal region that formed a solvent-exposed patch located on the side of Sso7d opposite to its DNA interface. Therefore, it was hypothesized that the N-terminal patch of Sso7d might interact with other chromatin proteins with the modulation by differential PTMs, as found with eukaryotic histones (Johnson et al., 2019).

Previous studies have established a potential epigenetic-like mechanism of methylation of Cren7 and Sul7d functioning in the environment adaptation of *Sulfolobus*, possibly through modulating their interactions with other (chromatin) proteins (Johnson et al., 2019). Whether the PTMs are directly involved in the DNA interactions of Cren7 and Sul7d remained unclear prior to this study. In the present study, we showed that methylation significantly affected Cren7, but not Sis7d, in their ability to bind DNA and to constrain negative DNA supercoils. Strikingly, methylated Cren7 was drastically less able to form oligomers in the absence of DNA or to mediate intermolecular DNA bridging but remained efficient in facilitating intramolecular DNA condensation. Single-site glutamine substitution of four lysine residues (K24, K31, K42, and K48) individually identified different roles for methylation at these sites in modulating Cren7-DNA interactions. Our results suggest the presence of a potential epigenetic-like mechanism that regulates chromatin structure through dynamic Cren7 methylation, in crenarchaea.

## Materials and Methods

### Growth of Organisms

All *Sulfolobus* strains were grown in SCVy medium (Peng et al., 2009) supplemented with 20 μg/ml uracil (SCVyU medium), a rich medium containing 0.2% sucrose, 0.2% casamino acid, 0.25% yeast and a mixed vitamin solution, at 65°C, 75°C or 85°C, with shaking at 150 r.p.m. *S. islandicus* E234 (Δ*pyrEF*,Δ*lacS*) is a generous gift from Professor Qunxin She at Shandong University. *S. islandicus* Δakmt is a deletion mutant of E234, constructed by Yindi Chu (Chu et al., 2016).

### Protein Overproduction and Purification

Native Cren7 and Sis7d was purified from *S. islandicus* (Guo et al., 2008). Cell lysates were prepared by sonication in Potassium Phosphate Buffer (30 mM, pH 6.6) with 50 mM NaCl and 10% Glycerol (buffer A). After centrifugation with 41000 r.p.m. for 1 h, the supernatant was applied to a HiTrap™ SP HP column (5 ml, GE) equilibrated in buffer A and eluted successively with 200 to 500 mM NaCl. Next, Proteins were loaded into a Superdex™ 75 column (10/300 GL, GE) equilibrated in buffer A. Fractions containing Cren7 and Sis7d then loaded onto RESOURCE S column (1 ml, GE) equilibrated in buffer A and eluted successively with 200 to 500 mM NaCl. Recombinant Cren7 and Sis7d were overproduced and purified as described previously (Lou et al., 2004; Zhang et al., 2010). Like native Cren7 and Sis7d, Cell lysates though centrifugation with 13000 r.p.m. for 30 min. The supernatant was heat-treated at 80°C for 20 min and centrifuged again. Then loaded sequentially into HiTrap™ SP HP column and Superdex 75 column. Protein concentrations were determined by the Lowry method using bovine serum albumin as the standard.

### Site-Directed Mutagenesis

Cren7 mutants were generated using the Fast Mutagenesis System (TransGen Biotech, China) with pET30a as the template (Zhang et al., 2010). All mutants were confirmed by DNA sequencing. The mutant constructs were transformed into *E. coli* BL21 (DE3) cells, and recombinant proteins were overproduced and purified as the wild-type protein.

### Mass Spectrometry and NanoLC–MS2/MS3 Data Acquisition

The extent methylation of Cren7 from *S. islandicus* was determined by RP-HPLC-C18-MS using the Orbitrap fusion Tribrid mass spectrometer (Thermo Fisher, United States) (Cao et al., 2018). The sites of methylation on Cren7 were identified by the Orbitrap Fusion Tribrid mass spectrometer fitted to an EASY-nLC 1000 UPLC system (Thermo Fisher, United States) (Cao et al., 2018). The raw data has been deposited in the China National Microbiology Data Center.^1^

### DNA Oligonucleotides

All oligonucleotides used in this study were synthesized commercially and purified by HPLC (Sangon, China) (Supplementary Table 1). To prepare double-stranded (ds) DNA fragments, complementary oligonucleotides were mixed in 20 mM Tris-HCl, pH 7.0, 100 mM NaCl and 1 mM EDTA, and the mixture was heated at 95°C for 5 min and subsequently cooled to room temperature in 2 h.

### Biolayer Interferometry Assays

BLI experiments were performed at 30°C at a speed of 1,000 rpm on the Octet Red96 system (ForteBio, United States). The biotin-labeled 30-bp dsDNA fragment D30 was prepared by annealing S30-F-biotin to S30-R at a molar ratio of 1:1.2. To determine the binding of wild-type and mutant Cren7 proteins to dsDNA, D30 was immobilized onto streptavidin biosensors about 0.3 nm level. Proteins were diluted in running buffer [20 mM Tris-HCl, pH 6.8, 100 mM KCl, 1 mM EDTA and 0.05% (v/v) Tween-20] to indicated concentrations. The biosensors were equilibrated for 60 s in the running buffer (baseline), and incubated for 60 s with proteins at different concentrations, followed by washing for 60 s in running buffer. The biosensors were regenerated by two cycles of sequential washing with 1 M NaCl for 10 s and the running buffer for 10 s. The association rate (*k*_*a*_), dissociation rate (*k*_*d*_) and the equilibrium dissociation constants (*K*_*D*_) were derived using a 1:1 binding model or steady state affinity model (Fortebio Data Analysis 8.5 Software). The binding kinetics for each protein was determined in triplicate.

### Nick-Closure Assays

Nick-closure assays were carried out as described previously (Zhang et al., 2017). Briefly, singly-nicked plasmid pBR322 was prepared by digestion with the nicking enzyme Nb. *Bpu*10I (Fermentas, United States) and purified. The nicked plasmid (0.3 μg) was incubated for 10 min at 25°C with various amounts of wild-type or mutant Cren7 in T4 ligase reaction buffer. After ligation with T4 DNA ligase (2 U; TransGen Biotech, China) for 2 min at 25°C, reaction was terminated with 50 mM EDTA and 0.5% SDS, and samples were deproteinized by treatment with protease K (2 mg/ml) for 2 h at 55°C. 20 μl of each sample (total volume of 30 μl) was then analyzed by electrophoresis in 1% agarose gel. The gels were imaged using BIORAD GELDOC2000 system after staining with M5 Gelred Plus (Mei5 Biotechnology, China).

### Chemical Cross-Linking

Chemical cross-linking assays were performed as described previously (Zhang et al., 2020). Each protein (3–5 μg) was cross-linked with 1 mM dithiobis (succinimidyl propionate) (DSP) for 30 min at 25°C in the presence or absence of salmon sperm DNA (3 and 5 μg, respectively) and subjected to 15% SDS-PAGE. Gels were stained with Rapid silver Staining Kit (Beyotime, China).

### DNA-Cellulose Pulldown Assays

Pulldown assays were conducted as described previously (Zhang et al., 2020). Briefly, 0.15 mg DNA-cellulose (Sigma-Aldrich), containing ∼750 ng dsDNA, was incubated for 30 min at room temperature with indicated amount of wild-type or mutant Cren7 and 500 ng pBR322 DNA in buffer A (10 mM HEPES, pH 7.6, 100 mM KCl, 2.5 mM MgCl_2_ and 0.1 mg/mL BSA). After washing with buffer A (500 μl), the bound pBR322 DNA was eluted in buffer B (10 mM Tris-HCl, pH 7.5, 5 mM EDTA, 200 mM NaCl and 0.2% SDS), deproteinized by treatment at 55°C for 1 h with protease K (1 mg/ml) and resolved by 1% agarose gel electrophoresis. Gels were stained with M5 Gelred Plus (Mei5 Biotechnology, China), and imaged using a Gel imager (BIORAD GELDOC2000).

### Atomic Force Microscopy

Samples for AFM visualization were prepared as described previously (Zhang et al., 2019). Briefly, native Cren7 from *S. islandicus* E234 was incubated for 40 min at room temperature with nicked pBR322 DNA (100 ng) at a monomer/bp ratio of 1:10 or 1:0.4 in a final volume of 20 μl. The complexes were fixed with 0.2% glutaraldehyde for 30 min at room temperature. After the addition of 50 mM Tris-HCl, pH 7.5, the samples were dialyzed to remove glutaraldehyde. Aliquots (5 μl) of the sample containing 2.5 mM MgCl_2_ were deposited onto freshly cleaved micas and allowed to stand for 5 min. The micas were rinsed three times with ddH_2_O and dried in a gentle stream of nitrogen gas. Visualization was performed under ambient conditions using a Nanoscope V Multimode-AFM instrument (Digital Instruments, Santa Barbara, CA, United States) in the ScanAsyst mode. Super-sharp silicon nitride tips SacnAsyst^®^ (Bruker, United States) with resonance frequency of ∼70 kHz were used at the scanning rate of ∼1 Hz.

## Results

### Growth Temperature Affects Methylation of Cren7 and Sis7d

**FIGURE 1:**
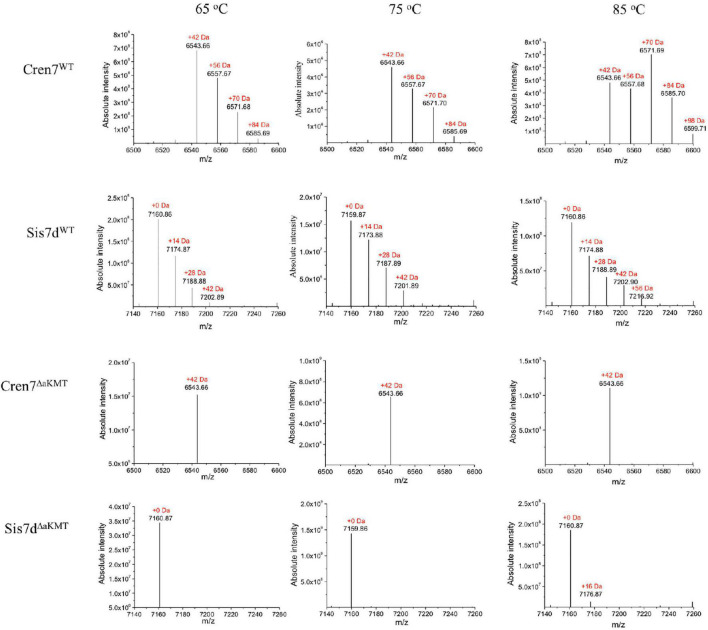
PTMs of Cren7 and Sis7d from *S. islandicus* grown at different temperatures. The molecular weights of Cren7 and Sis7d from the parent strain and ΔaKMT grown at different temperatures were measured by mass spectrometry. A cluster of peaks with a mass difference of 14 Da between adjacent peaks is shown.

Baumann et al. (1994) found that the methylation modifications of Sso7d associate with growth temperatures. To learn if growth temperature affected the methylation of Cren7 and Sis7d *in vivo*, we purified the two proteins from *S. islandicus* strain E234 grown at 65, 75, and 85°C in SCVyU medium to the mid-exponential phase and subjected them to mass spectrometry (MS). As shown previously (Cao et al., 2018), both proteins were methylated, exhibiting a cluster of peaks differing by 14 Da between adjacent peaks in the MS profiles (Figure 1). Notably, the methylation patterns of the two proteins were differently affected by temperature. Sis7d was methylated to a slightly greater extent as growth temperature increased. By comparison, Cren7 was slightly more methylated at 75°C than at 65°C, but it was drastically more extensively methylated at 85°C. Cren7 molecules with more methylation sites (up to four) became more predominant when the growth temperature was raised to 85°C. To determine if the observed changes in the level of methylation of the two proteins involved an increase in the number of sites of methylation, we purified native Cren7 and Sis7d from wild-type *S. islandicus* grown at 75 and 85°C and digested them with trypsin. Analysis of the resulting peptides by nanoLC-MS/MS on a high-resolution Orbitrap Fusion Tribrid instrument revealed that the modified peptides (Table 1 and Supplementary Figure 1) included nearly all of the methylated sites reported previously (Cao et al., 2018). Therefore, the difference of the two proteins in methylation at different growth temperatures resulted from changes in the level of methylation at individual sites, and the sites of methylation remained unchanged. It has been shown that the protein lysine methyltransferase aKMT is responsible for the methylation of both Cren7 and Sis7d in *Sulfolobus* (Chu et al., 2012, 2016). Since methylation of Sis7d at the three tested temperatures exhibited only slight changes whereas that of Cren7 at 85°C was far greater than at 75°C or 65°C, the two proteins are clearly different in temperature-affected susceptibility to methylation by aKMT *in vivo*.

**TABLE 1:** Non-redundant-modified peptides of Cren7 and Sis7d from *S. islandicus* E234 cells grown at 75 and 85°C*.

Protein	Peptide sequence	Methylation at 75°C	Max score	Methylation at 85°C	Max score
Cren7	^7^AVKVKTPAGK^16^	2 Methyl (K)	61.92	3 Methyl (K)	46.88
	^10^VKTPAGK^16^	2 Methyl (K)	36.33	2 Methyl (K)	48.06
	^10^VKTPAGKE^17^	Methyl (K)/Methyl (K)	38.08/34.14	2 Methyl (K)/Methyl (K)	38.71/35.86
	^12^TPAGKEAE^19^	Methyl (K)	29.89	Methyl (K)	29.83
	^24^KVWALAPK^31^	Methyl (K)	40.05	2 Methyl (K)/Methyl (K)	40.41/40.99
	^25^VWALAPK^31^	Methyl (K)	49.49	Methyl (K)	52.01
	^38^IGLFKDPETGK^48^	Methyl (K)	35.56	Methyl (K)	23.33
Sis7d	^2^TTVKFK^7^	Methyl (K)/2 Methyl (K)	28.27/34.15	Methyl (K)/2 Methyl (K)	20.9/34.26
	^55^LLQMLEK^61^	Methyl (K)	50.63	Methyl (K)	31.08
	^54^ELLQMLEK^61^	Methyl (K)	55.77	Methyl (K)	55.69
	^55^LLQMLEKQK^63^	Methyl (K)	51.42	Methyl (K)	38.57
	^29^MISFTYDEGGGK^40^	Methyl (K)	50.96	Methyl (K)	37.02

*All spectra have been manually inspected.

### Lysine Methylation Influences the DNA Binding Affinity of Cren7 and Not That of Sis7d

To test whether methylations would affect the interaction of Cren7 and Sis7d with DNA, we first compared the DNA binding affinities of the two proteins in a methylated form with those in an un-methylated form. Recombinant Cren7 and Sis7d overproduced in *E. coli*, denoted rCren7 and rSis7d, respectively, contained no modifications (Supplementary Figure 2). Native Cren7 and Sis7d were purified from *S. islandicus* E234 cells grown at 75°C. Cren7 from *S. islandicus* (Cren7^WT^) was both Nt-acetylated and methylated while that from *S. islandicus* ΔaKMT (Cren7^ΔaKMT^) was only Nt-acetylated. Sis7d from E234 (Sis7d^WT^) was methylated whereas that from ΔaKMT (Sis7d^Δ^ ^aKMT^) was unmodified. The binding affinities of these proteins to a 30-bp random dsDNA fragment (D30) were determined by BLI assays. As shown in Table 2, Sis7d^WT^ and Sis7d^ΔaKMT^ had similar apparent dissociation constants (*K*_*D*_ values of 1.64 ± 0.49 and 1.37 ± 0.41 μM, respectively), indicating that methylation did not measurably affect the binding of Sis7d to dsDNA. In contrast, the *K*_*D*_ of Cren7^WT^ (0.45 ± 0.05 μM) was approximately 2.6 fold as high as that of Cren7^ΔaKMT^ (0.17 ± 0.05 μM), suggesting the DNA was bound significantly less tightly by methylated Cren7 than the un-methylated one. The lower DNA binding affinity of Cren7^WT^, as compared to that of Cren7^ΔaKMT^, was due to the combination of a slightly decreased association rate and an ∼2-fold increased dissociation rate. Therefore, methylation of Cren7 resulted primarily in a decrease in the stability of the Cren7-DNA complex. It is noticed that Nt-acetylation did not appear to affect the binding of Cren7 to DNA since the *K*_*D*_ of rCren7 (0.13 ± 0.01 μM), which agrees well with the previous surface plasmon resonance data (Zhang et al., 2010), was close to that of Cren7^ΔaKMT^. Sis7d^ΔaKMT^ and rSis7d were also similar in DNA binding affinity with the *K*_*D*_ values of 1.37 ± 0.41 and 1.04 ± 0.30 μM, respectively. These results showed that protein methylation significantly affects DNA binding by Cren7 but not that by Sis7d. Therefore, we focused on the interaction of Cren7 with DNA in the subsequent analyses.

**TABLE 2:** Kinetic analysis of DNA binding by Cren7 and Sis7d from different strains*.

Protein	Binding affinity, *K*_*D*_ (M)	Association rate constant, *k*_a_ (M^–1^S^–1^)	Dissociation rate constant, *k*_d_ (S^–1^)
Cren7^WT^	4.46 ± 0.49 × 10**^–^**^7^	1.39 ± 0.16 × 10^5^	6.24 ± 1.21 × 10**^–^**^2^
Cren7^ΔaKMT^	1.65 ± 0.53 × 10**^–^**^7^	1.82 ± 0.40 × 10^5^	3.00 ± 0.04 × 10**^–^**^2^
rCren7	1.25 ± 0.13 × 10**^–^**^7^	1.20 ± 0.04 × 10^5^	1.50 ± 0.16 × 10**^–^**^2^
Sis7d^WT^	1.64 ± 0.49 × 10**^–^**^6^	1.28 ± 0.05 × 10^5^	2.09 ± 0.55 × 10**^–^**^1^
Sis7d^ΔaKMT^	1.37 ± 0.41 × 10**^–^**^6^	1.28 ± 0.06 × 10^5^	1.74 ± 0.44 × 10**^–^**^1^
rSis7d	1.04 ± 0.30 × 10**^–^**^6^	1.98 ± 0.83 × 10^5^	1.89 ± 0.43 × 10**^–^**^1^
K24Q	2.17 ± 0.41 × 10**^–^**^7^	2.49 ± 0.11 × 10^5^	5.37 ± 0.88 × 10**^–^**^2^
K31Q	1.03 ± 0.20 × 10**^–^**^7^	2.55 ± 0.17 × 10^5^	2.60 ± 0.36 × 10**^–^**^2^
K42Q	1.12 ± 0.38 × 10**^–^**^6^	1.13 ± 0.06 × 10^5^	1.28 ± 0.49 × 10**^–^**^1^
K48Q	1.99 ± 0.38 × 10**^–^**^7^	1.90 ± 0.11 × 10^5^	3.75 ± 0.49 × 10**^–^**^2^

*Each number is an average of three independent experiments.

### Protein Methylation Prevents DNA Bridging and Not DNA Packaging by Cren7

**FIGURE 2:**
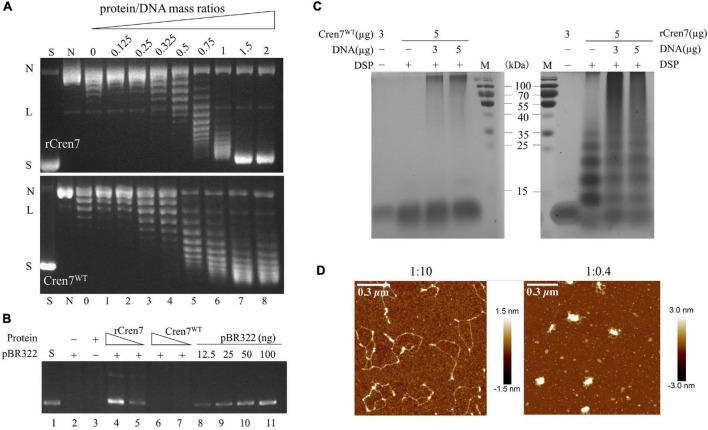
Effect of methylation on DNA packaging by Cren7. **(A)** The ability of Cren7^WT^ and rCren7 to constrain negative DNA supercoils. The singly-nicked DNAs after closure by T4 ligase were analyzed by agarose gel electrophoresis. S, negatively supercoiled pBR322; N, single-nicked pBR322; L, linear pBR322; lanes 0–8, pBR322 ligated in the presence of Cren7 at the protein/DNA mass ratios of 0, 0.125, 0.25, 0.325, 0.5, 0.75, 1, 1.5, and 2, respectively. **(B)** Detection of pBR322 DNA associated with DNA-cellulose by agarose gel electrophoresis. The dsDNA-cellulose pulldown assays were performed as described in Materials and Methods. After removal of unbound protein and pBR322 DNA by centrifugation, the pellet was resuspended in elution buffer. Aliquots of the eluted DNA were subjected to agarose gel electrophoresis. Lane 1, supercoiled pBR322 DNA (50 ng); lane 2, no protein added; lane 3, no pBR322 DNA added; lanes 4–5, rCren7 (10 and 5 μM) were added to the mixture of pBR322 DNA (500 ng) and DNA-cellulose (∼750 ng DNA); lanes 6–7, Cren7^WT^ (10 and 5 μM) were added; lanes 8–11, pBR322 DNA (12.5, 25, 50, and, 100 ng, respectively). **(C)** Chemical crosslinking of Cren7^WT^ and rCren7 in the absence or presence of salmon sperm DNA with or without 1 mM DSP. **(D)** AFM images of the Cren7^WT^-DNA complexes crosslinked with 0.2% glutaraldehyde. The protein/DNA ratios (in monomers/base pair) are indicated.

Recombinant Cren7 is capable of constraining negative DNA supercoils (Guo et al., 2008) and mediating DNA bridging and compaction (Zhang et al., 2020). These properties are consistent with the proposed role of the protein in chromosomal DNA organization. In this study, we determined if methylation would affect DNA packaging by Cren7 by comparing Cren7^WT^ and rCren7 in DNA topology and bridging assays. We found that Cren7^WT^ was about a half as efficient as rCren7 in constraining negative DNA supercoils in nick closure assays (Figure 2A), presumably because the former was less well bound to DNA than the latter. We then tested the ability of the two proteins to bridge DNA in DNA-cellulose pulldown assays. In the assays, pBR322 DNA (4,361 bp) was incubated with dsDNA-cellulose in the presence of rCren7 or Cren7^WT^, and the amounts of pBR322 DNA were pulled down with the resin and quantified by agarose gel electrophoresis (Figure 2B). As revealed previously (Zhang et al., 2020), ∼18 (∼90 ng) and 35% (∼175 ng) of the input pBR322 DNA were pulled down at rCren7 concentrations of 5 and 10 μM, respectively. By comparison, pBR322 DNA was barely detected (<10%) in the mixture containing 10 μM Cren7^WT^, suggesting that methylation substantially prevented Cren7 from mediating intermolecular DNA bridging.

The ability of recombinant Cren7 to mediate bridging appears to be correlated with that of the protein to form oligomers readily in solution (Zhang et al., 2020). To determine if methylation would affect the ability of Cren7 to oligomerize, we cross-linked both the methylated and unmethylated Cren7 with dithiobis (succinimidyl propionate) (DSP), a 1.2-nm cross-linker reactive toward amino groups in protein. Again, rCren7 formed oligomers in the absence of DNA and aggregates (may be different interaction forms to oligomers) in the presence of DNA, that could barely enter the gel, as reported previously (Figure 2C; Zhang et al., 2020). Although the nature of the aggregates is unclear, they appear to be assemblages of DNA-bound Cren7, as revealed by AFM (see below). By contrast, Cren7^WT^ was far less efficiently crosslinked with significantly more protein remaining in a monomeric form either in the presence or absence of DNA. Although methylation of 3–4 lysine residues on average in a Cren7 molecule would presumably reduce the efficiency of cross-linking of the protein with DSP, an amino group-coupling crosslinker, the drastic reduction observed in the amounts of the crosslinked products of Cren7^WT^ strongly suggests that lysine methylation inhibits the oligomerization of the protein in solution. This provides an interpretation for the observation that Cren7^WT^ failed to mediate intermolecular DNA bridging. Notably, however, once bound to DNA, Cren7^WT^ was crosslinked to form aggregates (Figure 2C). It appears that DNA-bound Cren7 monomers were aligned such that they were more readily crosslinked than when they were free in solution. To further explore the behavior of Cren7^WT^ on DNA, we cross-linked the protein in the presence of nicked pBR322 DNA with 0.2% glutaraldehyde and visualized the Cren7^WT^-DNA complexes by AFM. As shown in Figure 2D, the crosslinked Cren7^WT^-DNA complexes superficially resemble those of the rCren7-DNA complexes (Zhang et al., 2020). At the protein/DNA ratio (in monomer/bp) of 1:10, a physiologically relevant ratio, highly condensed core-like structures, which appeared to form along the DNA strand, presumably, as a result of lateral interaction between adjacently bound Cren7 monomers (Figure 2D). When the protein/DNA ratio increased to 1:0.4, the circular DNA was further condensed by Cren7 into a highly compacted structure with only one single condensed core surrounded by several small loops, as described for the rCren7-DNA complex formed under similar conditions (Zhang et al., 2020). These results, taken together, showed that protein methylation reduces both supercoil constraining and DNA bridging, but does not affect DNA packaging by Cren7.

### Methylation of Various DNA-Contacting Lysines Serves Different Roles in Cren7-DNA Interaction

**FIGURE 3:**
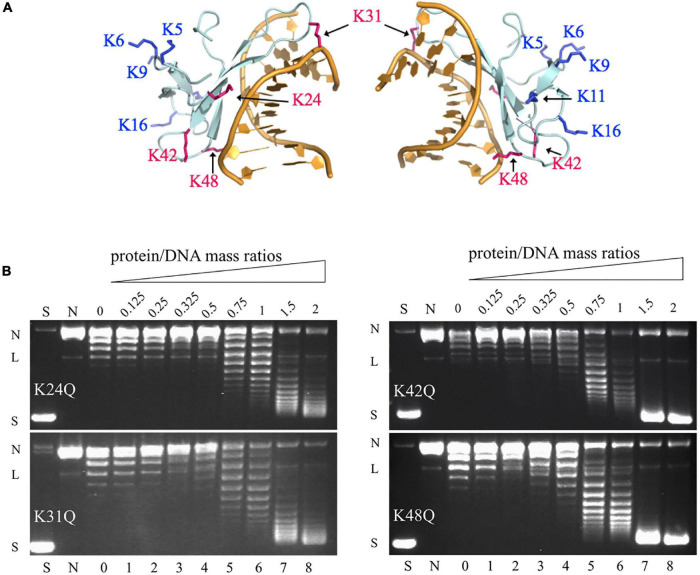
The ability of the mutant Cren7 proteins in constraining DNA supercoils. **(A)** Location of the methylated lysine residues on Cren7 in complex with an 8-bp DNA (PDB code: 3LWH). Four lysine residues (K24, K31, K42, and K48), either in direct contact with or in proximity to the DNA duplex, are shown in red, and the remaining lysine residues are shown in blue. **(B)** The ability of Cren7 mutants to constrain negative DNA supercoils. S, negatively supercoiled pBR322; N, single-nicked pBR322; lanes 0–8, topoisomers of pBR322 ligated in the presence of Cren7 mutants individually at protein/DNA mass ratios of 0, 0.125, 0.25, 0.325, 0.5, 0.75, 1, 1.5, and 2, respectively. L, linear pBR322.

Among the methylation sites of Cren7, K24, K31, K42, and K48 are located at the protein-DNA interface in the crystal structures of the Cren7-DNA complexes (Figure 3A; Zhang et al., 2010, 2019). To examine if methylation of these lysine residues would influence the interaction of Cren7 with DNA, we conducted site-directed mutation analysis for each of the four residues. It has been generally accepted that monomethylation slightly affects the hydrophobic character and size of the modified residue, but not change the overall charge (Musselman et al., 2012). Leucine and methionine (Hyland et al., 2011) and glutamine (Lu et al., 2013) have been used to mimic the methylated lysine, respectively. We chose glutamine to replace the lysine residues in our experiments because the polarity of the methylated lysines was supposed to function in the interactions between Cren7 and the phospho-ribose backbones of DNA. The resulting mutant proteins, i.e., K24Q, K31Q, K42Q, and K48Q, were produced recombinantly in *E. coli*, and then compared with rCren7 in their ability to bind DNA, constrain supercoils and mediate DNA bridging. We found that all of the four mutants, except for K31Q, bound less well to the 30-bp dsDNA fragment D30 than rCren7 in BLI assays (Table 2 and Supplementary Figure 3). Since substitution of Ala31 for K31 resulted in a large decrease (∼5 fold) in DNA binding affinity (Zhang et al., 2015), the long side chain rather than the positive charge here might be of importance in the Cren7-DNA interactions. K24Q and K48Q were ∼1.7 and 1.6-fold less efficient than in DNA binding than rCren7, respectively, as a result of an increase in both association and dissociation rates. K42Q showed the largest decrease (∼9.0 folds) in DNA binding affinity, primarily due to an 8.5-fold increase in dissociation rate, suggesting the critical importance of a positive charge at this position for the stability of the Cren7-DNA complex.

Among the four mutant proteins, K24Q and K31Q were significantly less efficient as compared to rCren7 (Figure 2A) in constraining DNA supercoils (Figure 3B). Since K24Q and K31Q were only slightly different from rCren7 in DNA binding, the positive charge of the two residues may play a role in facilitating local DNA distortion by Cren7. On the other hand, K48Q was similar to rCren7 in both DNA binding and supercoil constraining. Notably, K42Q constrained supercoils as well as rCren7 although the former had a much lower affinity for DNA than the latter. Therefore, it appears that K42 was not involved in the Cren7-induced DNA distortion. These results suggest that the ability of Cren7 to constrain DNA supercoils is not directly related to the affinity of the protein to DNA.

**FIGURE 4:**
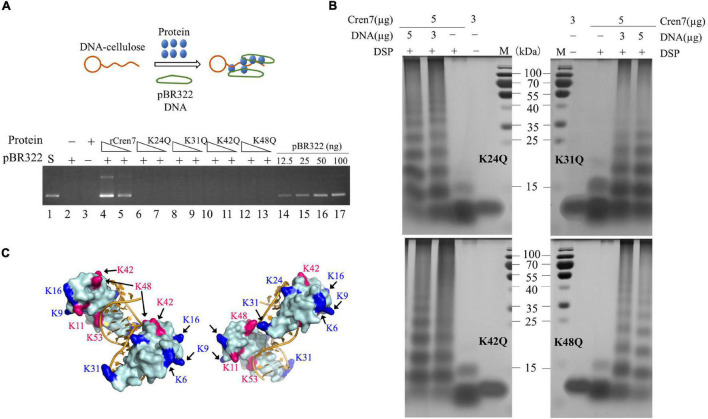
DNA-bridging and protein oligomerization of the mutant Cren7 proteins. **(A)** A diagram showing dsDNA-cellulose pulldown assays (upper panel). Double-stranded DNA cellulose was incubated with wild-type or mutant Cren7 and pBR322 DNA. Unbound protein and pBR322 DNA were removed by centrifugation. The efficiencies of the Cren7 mutants in mediating intermolecular DNA bridging were measured by DNA-cellulose pull-down assays (bottom panel). Lane 1, supercoiled pBR322 DNA (50 ng); lane 2, no protein added; lane 3, no pBR322 DNA added; lane 4–5, rCren7 (10 and 5 μM, respectively) were added to the mixture of pBR322 DNA (500 ng) and DNA-cellulose (∼750 ng DNA); lanes 6–7, Cren7^K24Q^ (10 and 5 μM, respectively); lanes 8–9, Cren7^K31Q^ (10 and 5 μM, respectively); lanes 10–11, Cren7^K42Q^ (10 and 5 μM, respectively); lanes 12–13, Cren7^K48Q^ (10 and 5 μM, respectively); lane 14–17, pBR322 DNA (12.5, 25, 50, and 100 ng, respectively). An aliquot of each sample was subjected to agarose gel electrophoresis. **(B)** Chemical crosslinking of the Cren7 mutants in the absence or presence of salmon sperm DNA with or without 1 mM DSP. **(C)** Locations of lysine residues involved in intermolecular cross-linking (red) and the remaining methylated lysine residues are shown in blue. Structural model of a complex of two Cren7 molecules with a 12-bp dsDNA was constructed from the crystal structure of Cren7 in complex with an 18-bp DNA (PDB code: 6A2I).

All of the four mutant proteins were inefficient in mediating DNA bridging. Like Cren7^WT^ (Figure 2B), the mutant proteins were hardly able to mediate the pull-down of pBR322 DNA by DNA-cellulose in the DNA bridging assays (Figure 4A). By estimation, the DNA bridging activity of the mutant proteins were <10% of that of rCren7. As observed with Cren7^WT^, the mutant proteins were not efficiently crosslinked into oligomers in the absence of DNA, suggesting that methylation of even a single lysine residue affected Cren7 oligomerization (Figure 4B). Thus, the failure of the mutant Cren7 proteins to mediate DNA bridging correlates with their oligomerization behavior. As shown in Figure 4C, the four tested sites of methylation are located close to sites of intermolecular crosslinking on Cren7. Indeed, crosslinking between lysine pairs, such as K31/K48, K48/K42, and K48/K53, by disuccinimidyl suberate (DSS) were detected by nano-LC-MS/MS (Zhang et al., 2020). These results indicated that lysine methylation is involved in the control of Cren7 oligomerization.

### The Methylation Pattern of Cren7 Is Variably Conserved in Different Crenarchaeal Branches

**FIGURE 5:**
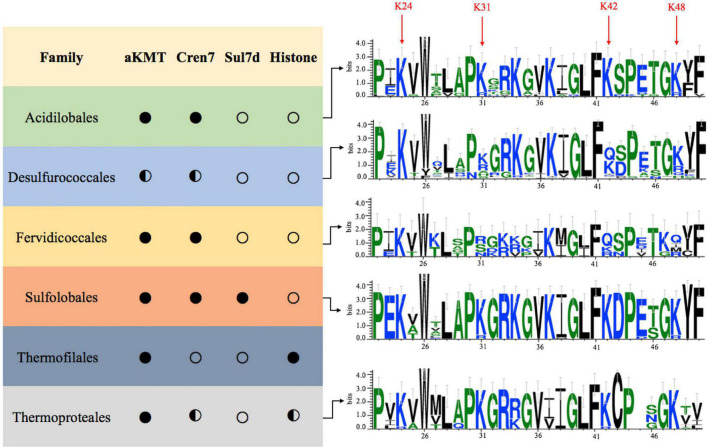
Conservation of the aKMT-catalyzed lysine methylation of Cren7 in the Crenarchaeota. Distributions of homologs of aKMT, Cren7, Sul7d and histone in each order of the Crenarchaeota are indicated with a filled circle (homologs are found in all species), half-filled circle (homologs are absent from some species) or hollow circle (no homologs are found in the order). The frequencies of amino acid residues in Cren7 homologs from each order are generated by Weblogo. The sequence numbers are labeled based on the sequence of Cren7 from *S. islandicus*.

The above results prompted us to examine if the observed Cren7 methylation is conserved among crenarchaea. As shown in Figure 5, both Cren7 and aKMT homologs are widespread and highly conserved in crenarchaea. Sequence alignment shows that K24, K31, K42 and K48 in Cren7 from *S. islandicus* are in general well conserved among the Cren7 homologs. K24 is conserved in all Cren7 homologues except for those from a couple of species of *Thermoproteaceae* which contain an arginine residue at the position corresponding to K24. K31, K42, and K48 are highly conserved, with infrequent arginine substitution, in three crenarchaeal orders, i.e., Sulfolobales, Acidilobales and Thermoproteales. However, lysine and arginine residues are found with similar frequencies at the position of K31 in Cren7 homologs from Desulfurococcales, and only arginine, serine or asparagine, but not lysine, is present at the position in those from Fervidicoccales. Similarly, a glutamine or arginine residue is present at the position of K48 in some of the Cren7 homologs from the orders Desulfurococcales and Fervidicoccales. Glutamine or arginine is also frequently found at the position corresponding to K42 in Cren7 homologs from these two orders. It should be noted that a glutamine or an arginine substitution would mimic a methylated or a non-methylated lysine residue. Given the known consequences of lysine methylation, glutamine or arginine substitution at any of the four lysine residues in Cren7 homologues would presumably affect the oligomerization of the protein as well as the protein-mediated DNA organization. Therefore, we concluded that the four lysine residues in Cren7 from *S. islandicus*, which undergo methylation by aKMT to varying degrees, are variably conserved among Cren7 homologues in crenarchaea, and replacement of the lysine residues with either a glutamine or an arginine, which would mimic different methylation states of lysine, often occurred in Cren7 homologues from some crenarchaeal branches.

## Discussion

Epigenetic modifications, such as DNA methylation and histone modifications, are known to serve important roles in the regulation of chromatin structure and gene expression (Bilmez et al., 2021). The tails of eukaryotic histones extend out of nucleosomes and are subjected to an array of modifications, e.g., methylation, acetylation and phosphorylation, which are key epigenetic mechanisms (Kadoch et al., 2016). However, archaeal histones, structural and functional homologs of eukaryotic histones, found in most Archaea, apparently lack post-translational modifications (Forbes et al., 2004). By contrast, Cren7 and Sul7d, two crenarchaeal chromatin proteins, both undergo extensive methylations on multiple lysine residues, and are thus potentially targets of epigenetic regulation. Indeed, recent work from Blum’s lab provided clues about the involvement of methylation of Cren7 and Sso7d in the regulation of transcription in *S. solfataricus* SARC strains (Payne et al., 2018; Johnson et al., 2019). In this study, we showed that both Cren7 and Sis7d from *S. islandicus* were slightly more methylated at 75°C (the optimal growth temperature) than at 65°C and, while the latter became still more methylated at 85°C (close to the maximal growth temperature), the former was methylated to a drastically greater extent. Therefore, the two structurally and biochemically similar chromatin proteins differ significantly in their susceptibility to methylation by aKMT at 85°C.

Our *in vitro* assays showed that methylation affected the binding of Cren7, and not Sis7d, to DNA, suggesting the potential role of the former in epigenetic regulation. The differences between methylated and unmethylated Cren7 proteins in DNA binding and supercoil constraining may suggest that the two forms of the protein may affect bound DNA differently in local conformation. Since methylated Cren7 formed protein aggregates on DNA as efficiently as unmethylated Cren7, intermolecular interaction between DNA-bound protein monomers does not appear to be affected by protein methylation. However, methylation significantly impaired the ability of Cren7 to bridge DNA strands. Therefore, Cren7 methylation may function in regulating gene expression by modulating local DNA distortion and cross-talks between sites distantly located on the genome.

Several lysine residues (K24, K31, K42, and K48) reside at the interface between Cren7 and its bound DNA. Glutamine substitution reveals that methylation at positions of K24 and K31 slightly reduced DNA binding by Cren7, but significantly decreased the ability of the protein to constrain negative supercoils, suggesting the involvement of methylation at these sites in local DNA deformation. Replacement of K42 with Q42 lowered the stability of Cren7-DNA complexes but displayed little effect on the ability of the protein to constrain supercoils. It is speculated that methylation of K42 was primarily responsible for the reduction in DNA binding by wild-type Cren7 as compared to that by rCren7. Methylation of K48 does not appear to be involved in DNA binding or supercoil constraining by Cren7. Intriguingly, like Cren7^WT^, glutamine substitution at each of these positions inhibited DNA bridging by Cren7. However, it should be noted that each of the four tested lysine residues is present in both methylated and un-methylated forms *in vivo*, and the observed effects of the K-to-Q mutation at these positions may represent predictions based on extreme circumstances. Therefore, with a varying level of methylation, Cren7 may serve a dynamic role in epigenetic regulation *in vivo*.

aKMT is widely distributed among Archaea. The key methylation sites of Cren7, i.e., K24, K31, K42, and K48, are conserved in most crenarchaeal orders, especially in Sulfolobales, Acidilobales and Thermoproteales, indicating the conservation of the lysine methylation pattern of Cren7. Therefore, it is tempting to speculate that epigenetic regulation through dynamic methylation of Cren7 at these sites is widely employed by crenarchaea. Intriguingly, K31, K42, and K48 in Cren7 homologs from Desulfurococcals and Feridicoccales are frequently replaced by glutamate or arginine, mimics of methylated or un-methylated lysines, respectively. The organisms may have evolved these Cren7 variants by converting an epigenetic site to a fixed genetic one to adapt to changes in habitats.

## Data Availability Statement

The original contributions presented in the study are included in the article/Supplementary Material, further inquiries can be directed to the corresponding author/s. The data presented in the study are deposited in the National Microbiology Data Center repository, accession number NMDCX0000118. Available at: https://nmdc.cn/resource/attachment/detail/NMDCX0000118.

## Author Contributions

ZZ initiated and designed the experiments. ZZ, LH, and CZ guided experimental design. ND, YChen, and YChu performed the experiments. ND, ZZ, and LH wrote and edited manuscript. All authors contributed to the article and approved the submitted version.

## Conflict of Interest

The authors declare that the research was conducted in the absence of any commercial or financial relationships that could be construed as a potential conflict of interest.

## Publisher’s Note

All claims expressed in this article are solely those of the authors and do not necessarily represent those of their affiliated organizations, or those of the publisher, the editors and the reviewers. Any product that may be evaluated in this article, or claim that may be made by its manufacturer, is not guaranteed or endorsed by the publisher.

## Footnotes

1
https://nmdc.cn/resource/attachment/detail/NMDCX0000118
