# Supplementary material for: Lysine Methylation Modulates the Interaction of Archaeal Chromatin Protein Cren7 With DNA
Source: Front Microbiol. 2022 Mar 3;13:837737. doi: 10.3389/fmicb.2022.837737 (PMC8927968; doi:10.3389/fmicb.2022.837737)
Supplement: Supplementary file 1 [file Table_1.DOCX]

Supplementary materials

Table S1. The gene sequences used in this study.

| Name | Sequence |
| --- | --- |
| S30-F-biotin | 5’ bio TAGAGCCCTAGTCTTCCAAACTAGCTACGC |
| S30-R | GCGTAGCTAGTTTGGAAGACTAGGGCTCTA |
| Cren7 | CATATGAGCAGCGGTAAAAAAGCGGTTAAAGTTAAAACCCCGGCGGGCAAAGAAGCGGAACTGGTTCCGGAAAAAGTTTGGGCGCTGGCGCCGAAAGGCCGTAAAGGCGTTAAAATCGGCCTGTTCAAAGATCCGGAAACCGGCAAATACTTCCGTCACAAACTGCCGGATGATTACCCGATCTAACTCGAG |
| K24Q | CATATGAGCAGCGGCAAAAAAGCGGTTAAAGTTAAAACCCCGGCGGGCAAAGAAGCGGAACTGGTTCCGGAACAGGTTTGGGCGCTGGCGCCGAAAGGCCGTAAAGGCGTTAAAATCGGCCTGTTCAAAGATCCGGAAACCGGCAAATACTTCCGTCACAAACTGCCGGATGATTACCCGATCTAACTCGAG |
| K31Q | CATATGAGCAGCGGTAAAAAAGCGGTTAAAGTTAAAACCCCGGCGGGCAAAGAAGCGGAACTGGTTCCGGAAAAAGTTTGGGCGCTGGCGCCGCAGGGCCGTAAAGGCGTTAAAATCGGCCTGTTCAAAGATCCGGAAACCGGCAAATACTTCCGTCACAAACTGCCGGATGATTACCCGATCTAACTCGAG |
| K42Q | CATATGAGCAGCGGCAAAAAAGCGGTTAAAGTTAAAACCCCGGCGGGCAAAGAAGCGGAACTGGTTCCGGAAAAAGTTTGGGCGCTGGCGCCGAAAGGCCGTAAAGGCGTTAAAATCGGCCTGTTCCAGGATCCGGAAACCGGCAAATACTTCCGTCACAAACTGCCGGATGATTACCCGATCTAACTCGAG |
| K48Q | CATATGAGCAGCGGCAAAAAAGCGGTTAAAGTTAAAACCCCGGCGGGCAAAGAAGCGGAACTGGTTCCGGAAAAAGTTTGGGCGCTGGCGCCGAAAGGCCGTAAAGGCGTTAAAATCGGCCTGTTCAAAGATCCGGAAACCGGCCAGTACTTCCGTCACAAACTGCCGGATGATTACCCGATCTAACTCGAG |


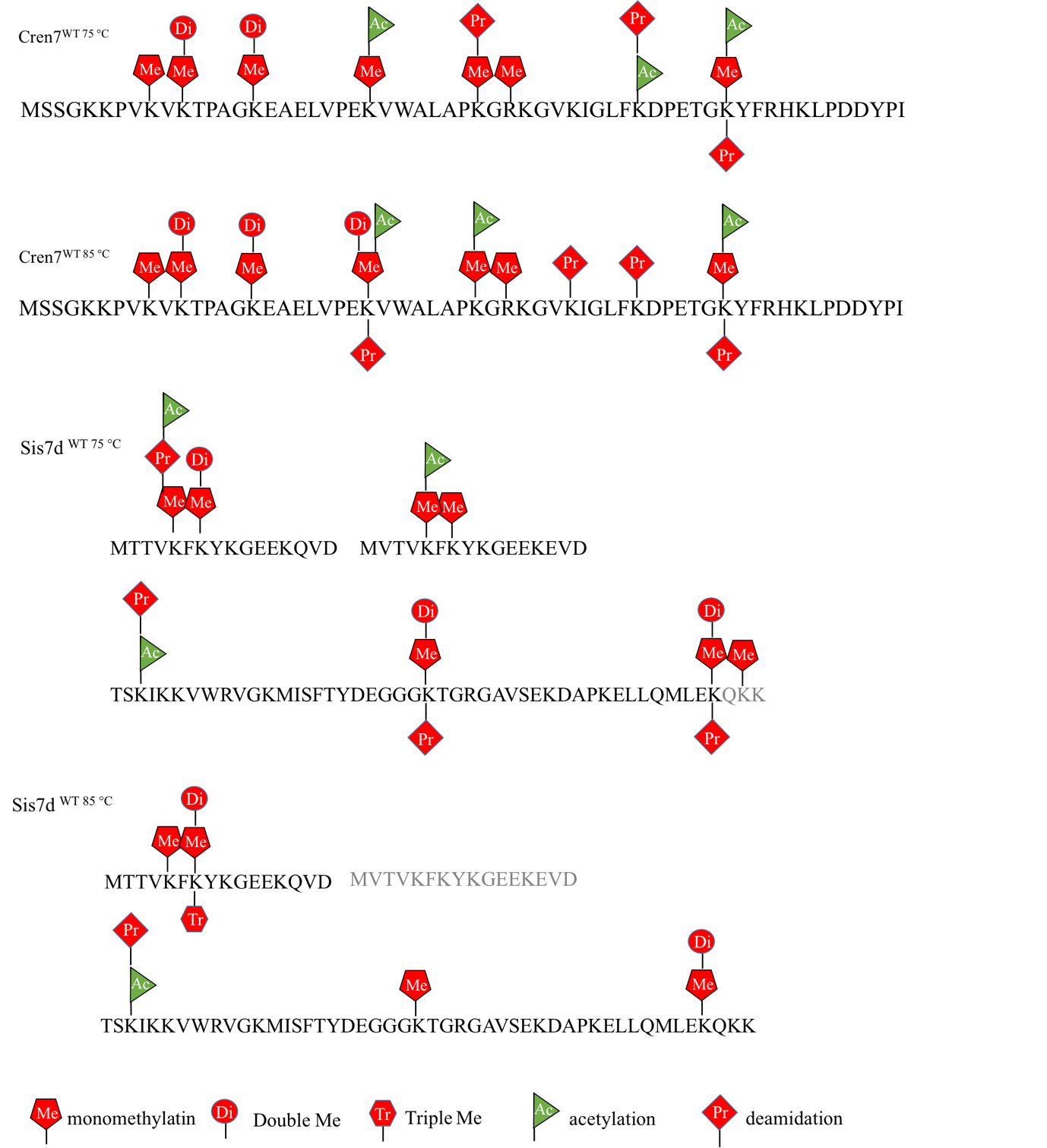


Figure S1. Sites and nature of the PTMs of Cren7 and Sis7d from *S. islandicus* grown at different temperatures. Unidentified peptides are in grey.


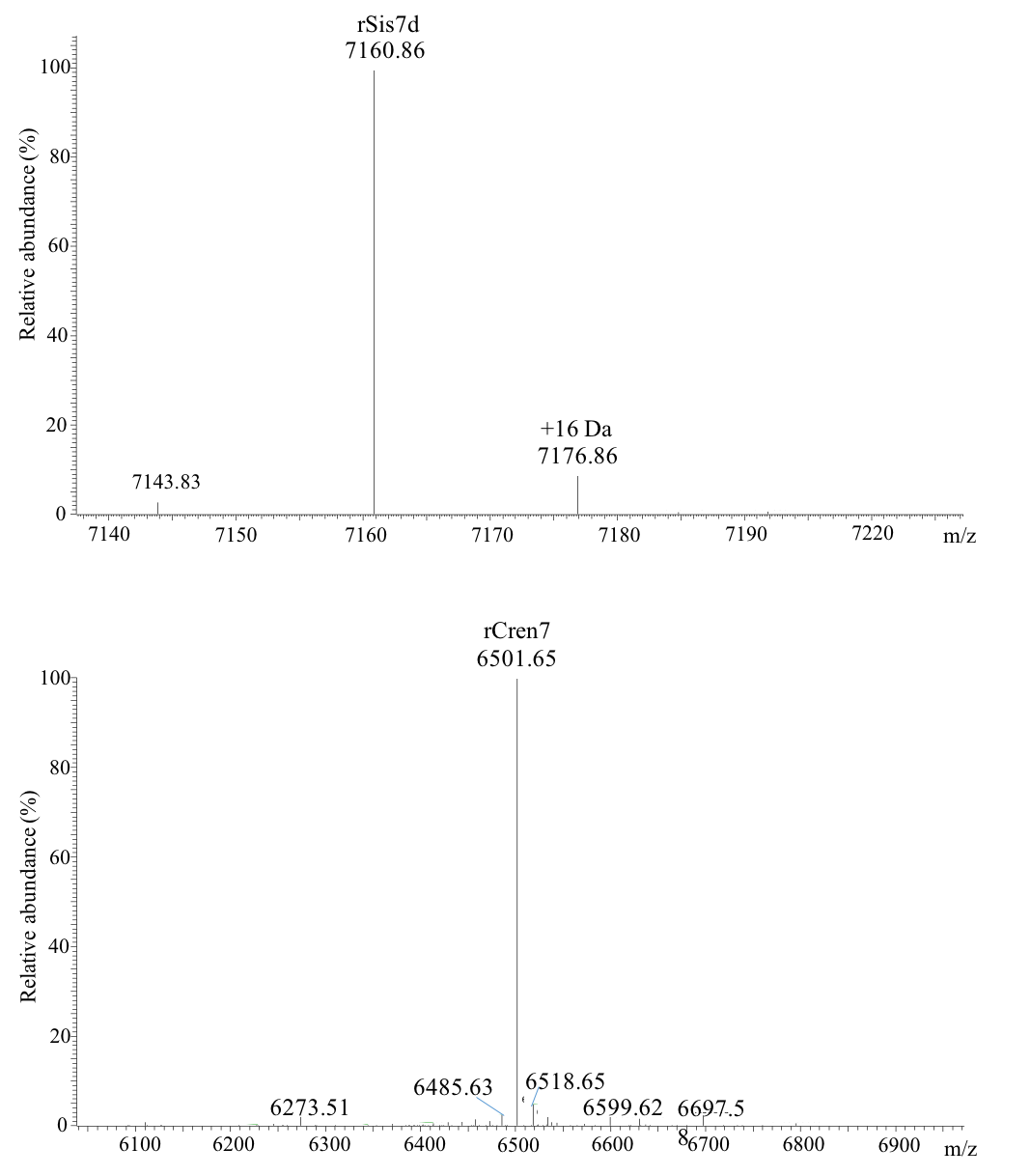
Figure S2. The molecular weights of recombinant Cren7 and Sis7d. The molecular weights of recombinant Cren7 and Sis7d overproduced in *E. coli* were measured by Mass spectrometry. The peak labelled as +16 Da indicates an oxidation event.


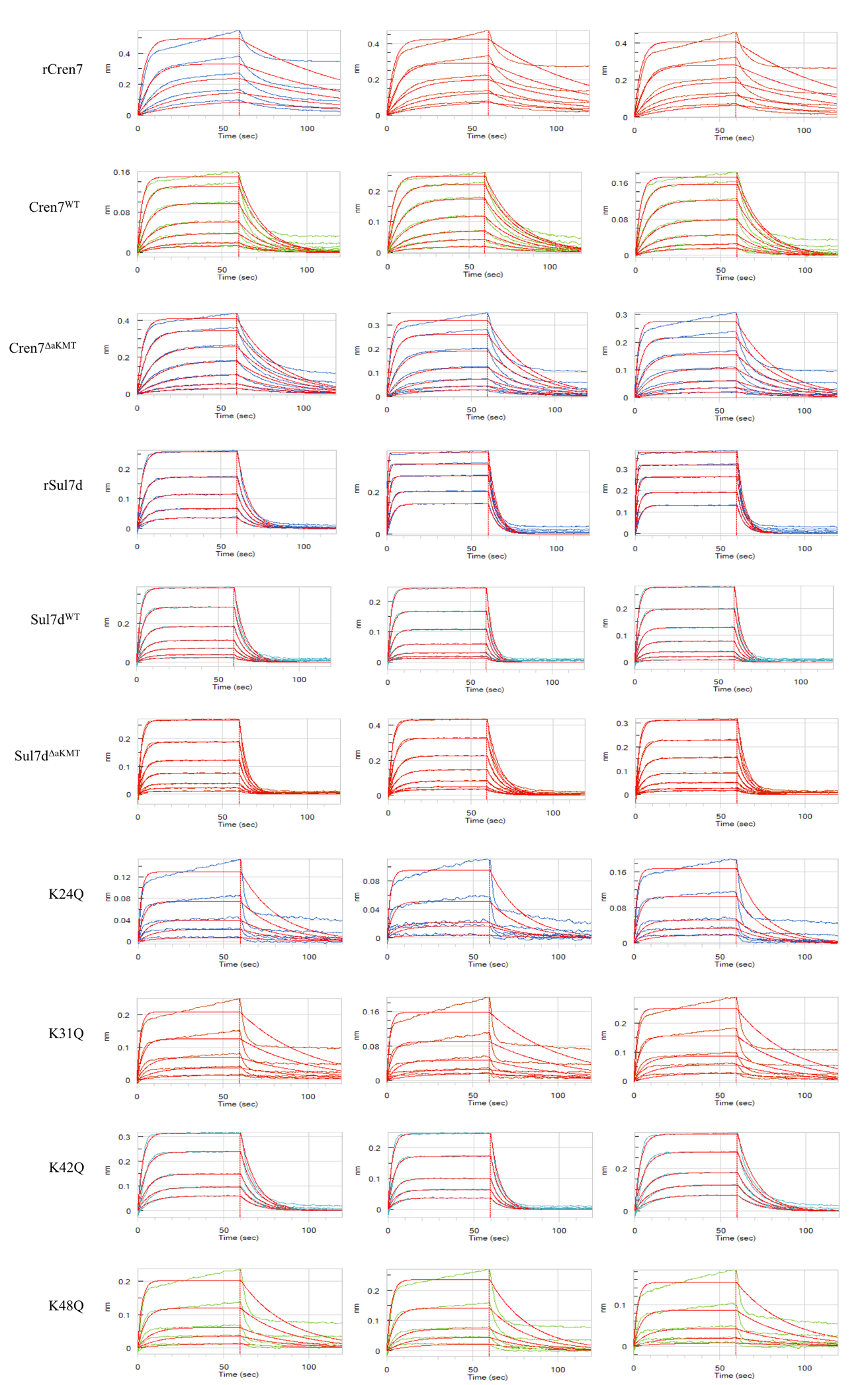
Figure S3. Kinetic analysis of DNA binding by Sis7d and Cren7. Each set of three diagrams represents three independent experiments.
